# Ursolic acid synergistically enhances the therapeutic effects of oxaliplatin in colorectal cancer

**DOI:** 10.1007/s13238-016-0295-0

**Published:** 2016-07-29

**Authors:** Jianzhen Shan, Yanyan Xuan, Qi Zhang, Chunpeng Zhu, Zhen Liu, Suzhan Zhang

**Affiliations:** 1 Department of Medical Oncology, The Second Affiliated Hospital, Zhejiang University School of Medicine, Hangzhou, 310009 China; 2 Cancer Institute of Zhejiang University School of Medicine, Hangzhou, 310009 China; 3 Department of Hepatobiliary and Pancreatic Surgery, The Second Affiliated Hospital, Zhejiang University School of Medicine, Hangzhou, 310009 China; 4 Department of Gastroenterology, The Second Affiliated Hospital, Zhejiang University School of Medicine, Hangzhou, 310009 China

**Keywords:** ursolic acid, colorectal cancer, oxaliplatin, signaling pathways

## Abstract

Oxaliplatin is a key drug in chemotherapy of colorectal cancer (CRC). However, its efficacy is unsatisfied due to drug resistance of cancer cells. In this study, we tested whether a natural agent, ursolic acid, was able to enhance the efficacy of oxaliplatin for CRC. Four CRC cell lines including SW480, SW620, LoVo, and RKO were used as *in vitro* models, and a SW620 xenograft mouse model was used in further *in vivo* study. We found that ursolic acid inhibited proliferation and induced apoptosis of all four cells and enhanced the cytotoxicity of oxaliplatin. This effect was associated with down-regulation of Bcl-xL, Bcl-2, survivin, activation of caspase-3, 8, 9, and inhibition of KRAS expression and BRAF, MEK1/2, ERK1/2, p-38, JNK, AKT, IKKα, IκBα, and p65 phosphorylation of the MAPK, PI3K/AKT, and NF-κB signaling pathways. The two agents also showed synergistic effects against tumor growth *in vivo*. In addition, ursolic acid restored liver function and body weight of the mice treated with oxaliplatin. Thus, we concluded that ursolic acid could enhance the therapeutic effects of oxaliplatin against CRC both *in vitro* and *in vivo*, which offers an effective strategy to minimize the burden of oxaliplatin-induced adverse events and provides the groundwork for a new clinical strategy to treat CRC.

## INTRODUCTION

Colorectal cancer (CRC) is the second leading cause of cancer-related deaths in the United States and the third most common cancer both in men and in women (Siegel et al., 2013). In China, it is the fifth most common cancer and also the fifth leading cause of cancer-related deaths. In the year 2012, there were 310,000 new CRC cases diagnosed and 150,000 deaths caused by CRC in China, and the number is still increasing (Chen et al., 2015). Although therapeutic advances had been made in the last decades, the prognosis of advanced CRC patients remains poor (Ciombor et al., 2015).

As a malignant disease, multiple signaling pathways involving cell survival and proliferation are frequently over-activated in CRC. For instance, the mitogen-activated protein kinase (MAPK) signaling pathway comprising RAS/RAF/MEK/ERK plays a key role in CRC. Activating mutations of RAS or RAF increase the signal flux and lead to the nuclear translocation of the final effector ERK1/2 and induction of downstream target genes (Hatzivassiliou et al., 2013). Genetic anomalies of the phosphatidylinositol-3 kinase (PI3K) pathway, such as PI3K amplification/mutation, AKT mutation, or PTEN loss, result in activation of this pathway, leading to tumorigenesis and have been implicated in the resistance of KRAS mutant CRC cells to MEK inhibition (Temraz et al., 2015). Moreover, increased signaling from the MAPK and the PI3K pathways also activates the IKK complex by crosstalking with the NF-κB signaling cascade, which facilitates the phosphorylation and subsequent proteosomal degradation of the NF-κB inhibitor, IκBα. NF-κB thus translocates to the nucleus and manifests its tumor promoting effects (Hassanzadeh, 2011). The aberrant activation of multiple signaling pathways in CRC makes it difficult for single target drugs to achieve satisfactory therapeutic effects (De Roock et al., 2010; Shimizu et al., 2012). Novel drugs with multiple targets are therefore urgently needed.

Oxaliplatin is a third-generation platinum compound used for the treatment of colorectal, gastric, and pancreatic cancers and is under clinical trials in ovarian, breast, and non-small cell lung cancers. It is typically administered together with 5-fluorouracil and folinic acid in a combination known as FOLFOX to treat CRC as palliative or adjuvant chemotherapy (Andre et al., 2009). Despite its favorable clinical efficacy, dose-limiting side effect (such as neuropathy) of oxaliplatin-based chemotherapy prevents administration of the full efficacious dosage and frequently leads to treatment withdrawal (Avan et al., 2015; Martinez-Balibrea et al., 2015). Evidence of liver damage in CRC patients following oxaliplatin treatment was also reported (Morris-Stiff et al., 2008; Nordlinger et al., 2013). It is therefore of paramount importance to develop novel drugs that could safely and effectively complement or enhance the therapeutic effects of oxaliplatin.

Ursolic acid (3β-hydroxy-urs-12-en-28-oicacid, UA) is a pentacyclic triterpenic acid found in a variety of natural plants, including the Chinese medicinal herbs such as *Hedyotic diffusa* and *Prunell avulgaris* (Wozniak et al., 2015). It exhibits a broad range of pharmacological effects such as anti-inflammatory, antiviral, antioxidant, hepatoprotective, etc. (Saravanan et al., 2006; Checker et al., 2012; Li et al., 2014) In addition, it has shown great promise in treating a number of cancers, such as lung cancer, hepatocellular carcinoma, colorectal cancer, prostate cancer, melanoma, and breast cancer (Gao et al., 2012; Prasad et al., 2011; Prasad et al., 2012; Shanmugam et al., 2013; Shishodia et al., 2003; Xavier et al., 2009; Xiang et al., 2015). More encouragingly, it has recently been pushed forward to enter clinical trials investigating its effects on insulin sensitivity (phase II study) and muscle function in human sarcopenia (phase II and III studies) (Ebert et al., 2015). We have previously shown that ursolic acid is capable of inhibiting the growth of a CRC cell line HT-29 by suppressing the MAPK signaling pathway (Shan et al., 2009; Shan et al., 2011). However, whether UA could enhance the therapeutic effects of oxaliplatin in CRC still remains largely unknown. Thus, in this study, the potential effects of ursolic acid to enhance the cytotoxicity of oxaliplatin were examined *in vitro* and in an *in vivo* CRC model, and the underlying molecular mechanisms were also investigated.

## RESULTS

### Ursolic acid inhibited the proliferation and induced apoptosis of CRC cells *in vitro*

**Figure 1:**
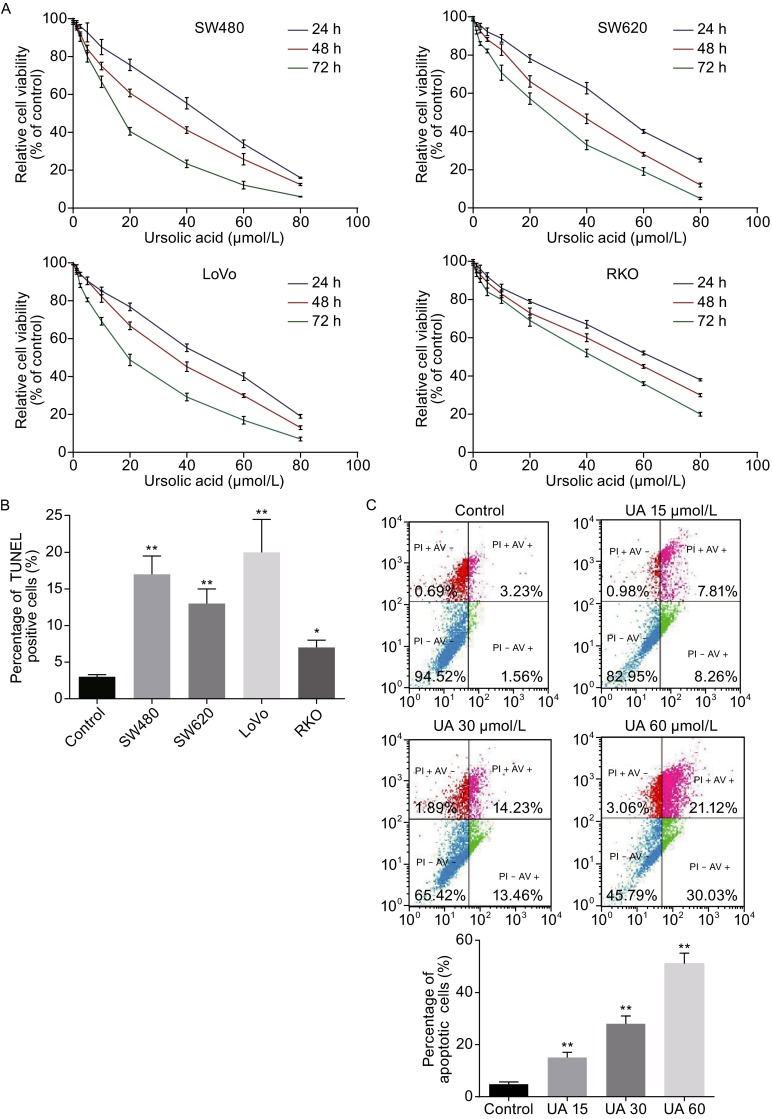

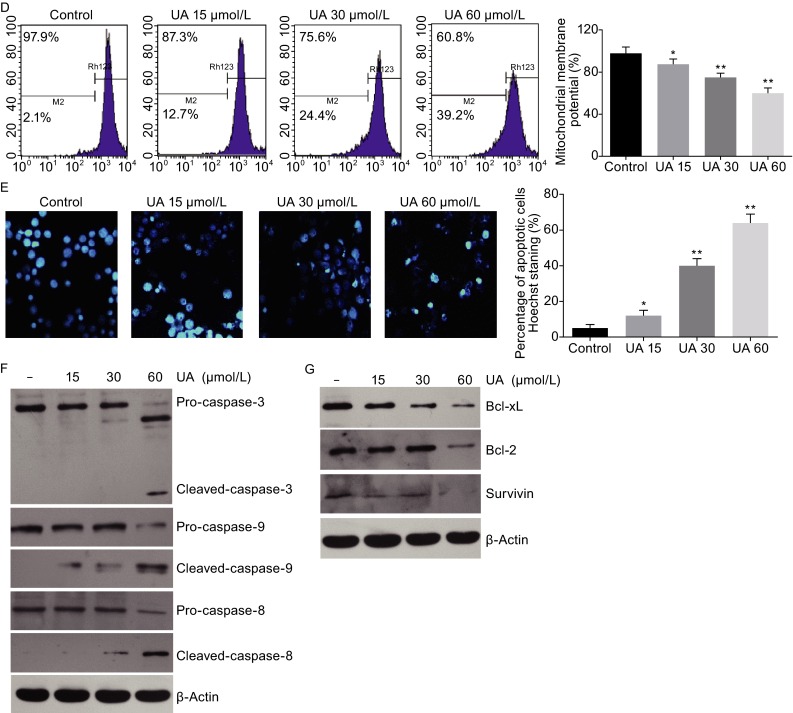
**Ursolic acid inhibited proliferation and induced apoptosis in human CRC cell lines**. (A) Ursolic acid inhibited proliferation of the four CRC cell lines in dose- and time-dependent manners as determined by MTT assay. (B) Ursolic acid induced apoptosis in four CRC cell lines as detected by TUNEL assay. Data represents the median values of triplicate experiments. (C–E) Ursolic acid induced apoptosis of SW620 as detected by AV/PI staining, mitochondrial membrane potential loss, and Hoechst 33342 staining. Quantitative analysis was performed for each method and the results were shown on the right, respectively. (F) Ursolic acid activated caspase cleavage in SW620 cells after 48 h treatment. (G) Ursolic acid inhibited the expression of Bcl-xL, Bcl-2, and survivin in SW620 cells

To identify the cytotoxicity of ursolic acid, we first examined whether ursolic acid could inhibit the proliferation of CRC by using several cell lines including SW480, SW620, LoVo, and RKO. As a result, ursolic acid suppressed proliferation in both dose- and time-dependent manners (Fig. 1A). The half minimal inhibitory concentration (IC_50_) values of ursolic acid in the four cell lines ranged from 16.76 to 48.12 μmol/L typically at 48 h (Table 1).

**Table 1:** IC_50_ values and statistical analyses of ursolic acid and oxaliplatin treatments in four CRC cell lines

Cell lines	IC_50_					
	UA (μmol/L)			OXL (μmol/L)	UA (μmol/L) + OXL (μmol/L)	Interaction
	24 h	48 h	72 h	48 h	48 h	
SW480	26.22 (21.09 to 33.13)	16.76 (13.53 to 22.43)	14.13 (10.45 to 20.07)	0.35 (0.28 to 0.39)	UA 5.21 (4.32 to 7.12) OXL 0.07 (0.05 to 0.13)	0.51 synergy
SW620	35.93 (30.26 to 42.66)	30.61 (25.89 to 37.13)	28.26 (24.13 to 33.09)	0.68 (0.45 to 0.87)	UA 9.03 (8.25 to 10.19) OXL 0.16 (0.11 to 0.21)	0.55 synergy
LoVo	22.77 (19.71 to 27.56)	20.16 (17.13 to 24.17)	16.78 (13.57 to 21.06)	0.41 (0.30 to 0.51)	UA 6.03 (5.25 to 7.03) OXL 0.10 (0.06 to 0.17)	0.54 synergy
RKO	61.03 (52.34 to 66.45)	48.12 (31.15 to 53.59)	38.83 (25.30 to 42.84)	0.79 (0.59 to 0.91)	UA 15.25 (9.15 to 16.15) OXL 0.29 (0.17 to 0.38)	0.68 synergy

We next examined the apoptosis of these CRC cells treated with ursolic acid (IC_50_ for 48 h) by TUNEL assay. Ursolic acid significantly induced apoptosis in all the four cell lines by around 2 to 10 times (Fig. 1B). We further used SW620 cells for characterization of apoptosis induced by ursolic acid. As expected, flow cytometry analysis revealed that ursolic acid induced apoptosis in a dose-dependent manner (Fig. 1C). In particular, ursolic acid-caused decrease of mitochondrial membrane potential, which was known to activate caspases and initiate apoptotic cascades was detected (Fig. 1D). Hoechst 33342 staining assay also confirmed the pro-apoptosis effect of ursolic acid (Fig. 1E). Consistently, activation of caspases were identified by immunoblotting in ursolic acid-treated SW620 cells, with dramatically increased cleavage of pro-apoptotic molecules caspase-3, -9, and -8 (Fig. 1F) as well as inhibited expression of anti-apoptotic molecules including Bcl-xL, Bcl-2, and survivin (Fig. 1G). Taken together, these results showed ursolic acid was able to induce apoptosis in CRC cells.

### Ursolic acid synergistically inhibited proliferation with oxaliplatin and enhanced oxaliplatin-induced apoptosis in CRC cells

**Figure 2:**
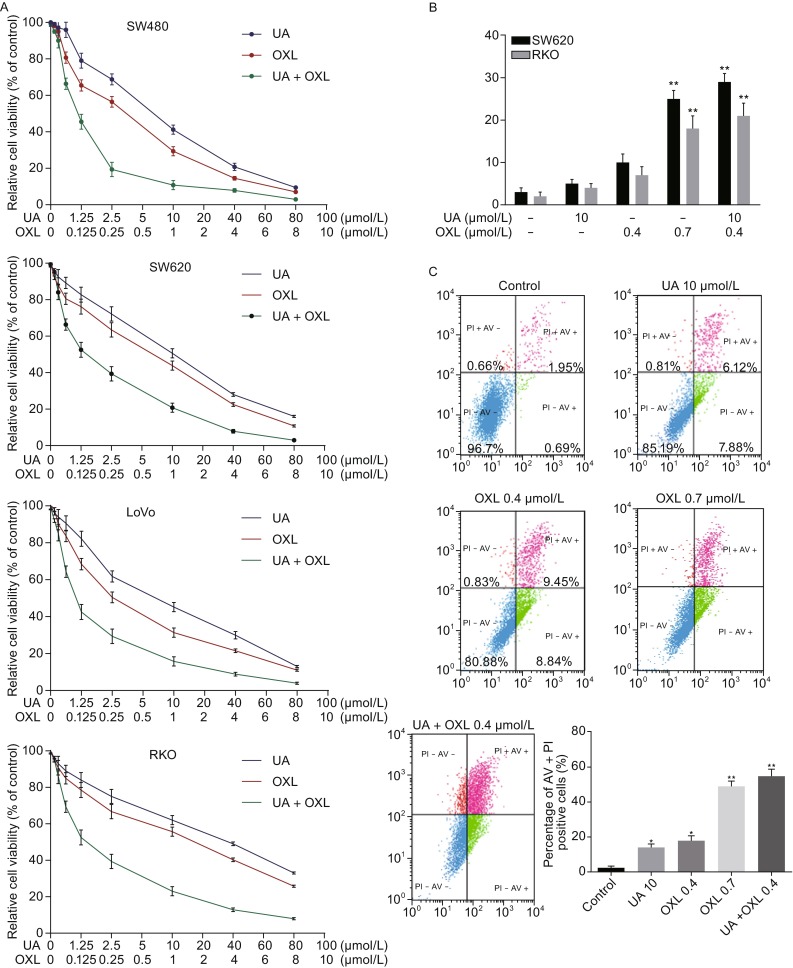

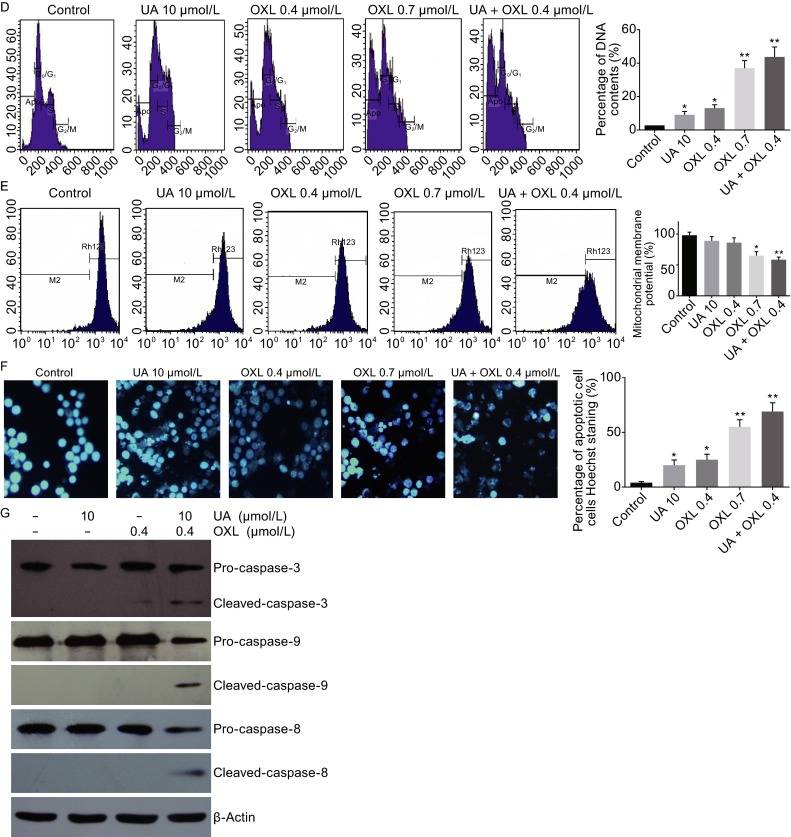
**Ursolic acid synergistically inhibited CRC cell growth with oxaliplatin and enhanced oxaliplatin-induced apoptosis in CRC cells**. (A) Synergistic inhibitory effects of ursolic acid and oxaliplatin against four CRC cell lines as determined by MTT assay. (B) Quantification of TUNEL positive SW620 and RKO cells after treating with ursolic acid or oxaliplatin alone or in combination for 48 h. (C–G) SW620 cells were treated with ursolic acid or oxaliplatin alone or in combination for 48 h and cell apoptosis was detected by flow cytometry using five different methods: AV/PI staining, DNA content measurement, mitochondrial membrane potential loss, and Hoechst 33342 staining, and immunoblotting of caspase cleavage. Quantitative analysis was performed for each method and the results were shown on the right, respectively

We further explored the efficacy of combination of ursolic acid and oxaliplatin in CRC cells. All the four cell lines were treated with various concentrations of ursolic acid and oxaliplatin alone or in combination for 48 h and cell proliferation was determined by MTT assay. The combination of ursolic acid and oxaliplatin showed more cytotoxic to all the four cell lines compared to either treatment alone (Fig. 2A). Drug combination index analysis indicated that ursolic acid had a synergistic effect with oxaliplatin in inhibiting the proliferation of all cell lines tested (Table 1).

Next, we assessed apoptosis in SW620 and RKO cells induced by ursolic acid and oxalipatin alone or in combination by TUNEL assay. We found that suboptimal dosages of ursolic acid (10 μmol/L) and oxaliplatin (0.4 μmol/L) induced less than 10% of apoptosis in SW620 cells. However when combined, the cell apoptosis rate increased significantly to about 30%, which was slightly better than the cytotoxic effect observed in higher dose of oxaliplatin treatment at 0.7 μmol/L (Fig. 2B). Similar results were also obtained in RKO cells.

We continued to assess apoptosis in SW620 cells by flow cytometry. Combination of the two agents showed a better pro-apoptosis effect than either agent alone by using the annexin V/propidium iodide staining (Fig. 2C) and the DNA content analysis (Fig. 2D). Consistently, reduction of mitochondrial membrane potential was more significant when SW620 cells were treated with the two agents simultaneously (Fig. 2E). This effect was also confirmed by Hochest 33342 staining assay (Fig. 2F). The cleavage of caspase-3, -8, -9 was also observed in the combination therapy (Fig. 2G).

### Ursolic acid alone and in combination with oxaliplatin suppressed the MAPK, PI3K/AKT and NF-κB signaling pathways

The MAPK, PI3K/AKT and NF-κB pathways are frequently activated in CRC contributing significantly to tumorigenesis and the resistance to chemotherapy. The capability of UA to inhibit these pathways in various tumors was well documented (Achiwa et al., 2013; He et al., 2015; Shanmugam et al., 2011; Li et al., 2012). However, it remains largely unknown whether it has similar inhibitory effects in CRC. We previously showed that ursolic acid inhibited the growth and induced apoptosis of HT-29 CRC cells by suppressing the phosphorylation of EGFR, ERK1/2, p38, and JNK of the MAPK signaling pathway (Shan et al., 2009). Here, we continued to investigate the potential underlying molecular mechanisms through which ursolic acid killed the CRC cells and enhanced the cytotoxic effects of oxaliplatin.

**Figure 3:**
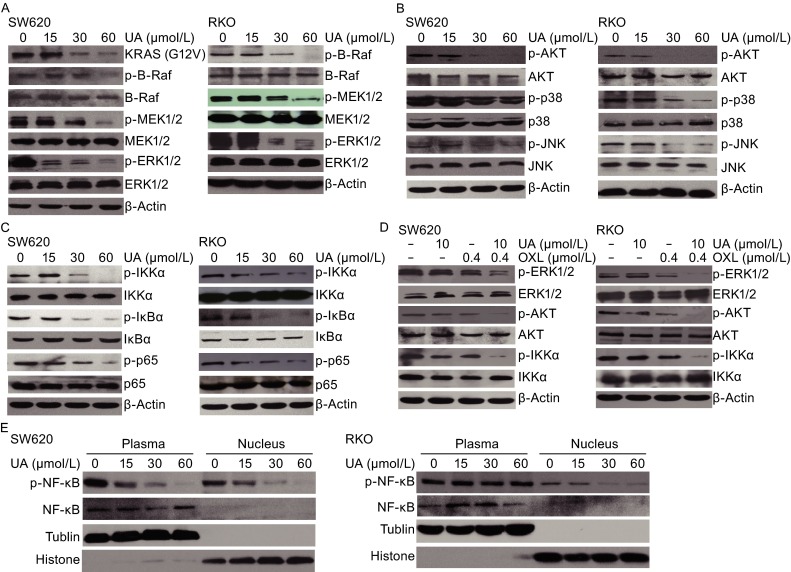
**MAPK, PI3K/AKT, and NF-κB pathways were affected by ursolic acid with or without oxaliplatin in SW620 and RKO cells**. (A) Ursolic acid inhibited protein phosphorylation of BRAF, MEK1/2, ERK1/2 of the MAPK signaling pathway in SW620 and RKO cells in a dose-dependent manner. (B) Ursolic acid inhibited protein phosphorylation of AKT, p38, and JNK in SW620 and RKO cells in a dose-dependent manner. (C) Ursolic acid inhibited protein phosphorylation of IKKα, IκBα, and p65 of the NF-κB signaling pathway in SW620 and RKO cells in a dose-dependent manner. (D) Ursolic acid inhibited p-p65 in nucleus in a dose-dependent manner in SW620 and RKO cells. (E) SW620 and RKO cells were treated with ursolic acid or oxaliplatin alone or in combination for 48 h and the phosphorylation status of ERK1/2, AKT, and IKKα was analyzed by Western blotting

SW620 and RKO cells were treated with different concentrations of ursolic acid for 48 h and we detected significant down-regulation of p-B-Raf, p-MEK1/2, p-ERK1/2, p-Akt, p-p38, and p-JNK as a dose-dependent manner without influence on their total proteins (Fig. 3A and 3B). In addition, NF-κB signaling was also affected by ursolic acid, with significant reduced phosphorylation of IKKα, IκBα, and p65 (Fig. 3C). Typically, ursolic acid significantly reduced p-p65 in the nucleus (Fig. 3D), suggesting a potential transcriptional inhibition of NF-κB target genes. Encouragingly, the combination of ursolic acid and oxaliplatin remarkably inhibited p-ERK1/2, p-Akt, and p-IKKα, which was much more significant compared to those treated with ursolic acid or oxaliplatin alone (Fig. 3E). These results suggested that ursolic acid was capable of inhibiting multiple critical kinases in tumor progression, and the facilitation of inhibition of these signaling pathways might also be responsible for the enhanced effects when combined with oxaliplatin.

### Synergistic antitumor activity of ursolic acid and oxaliplatin in SW620 xenograft mouse model

**Figure 4:**
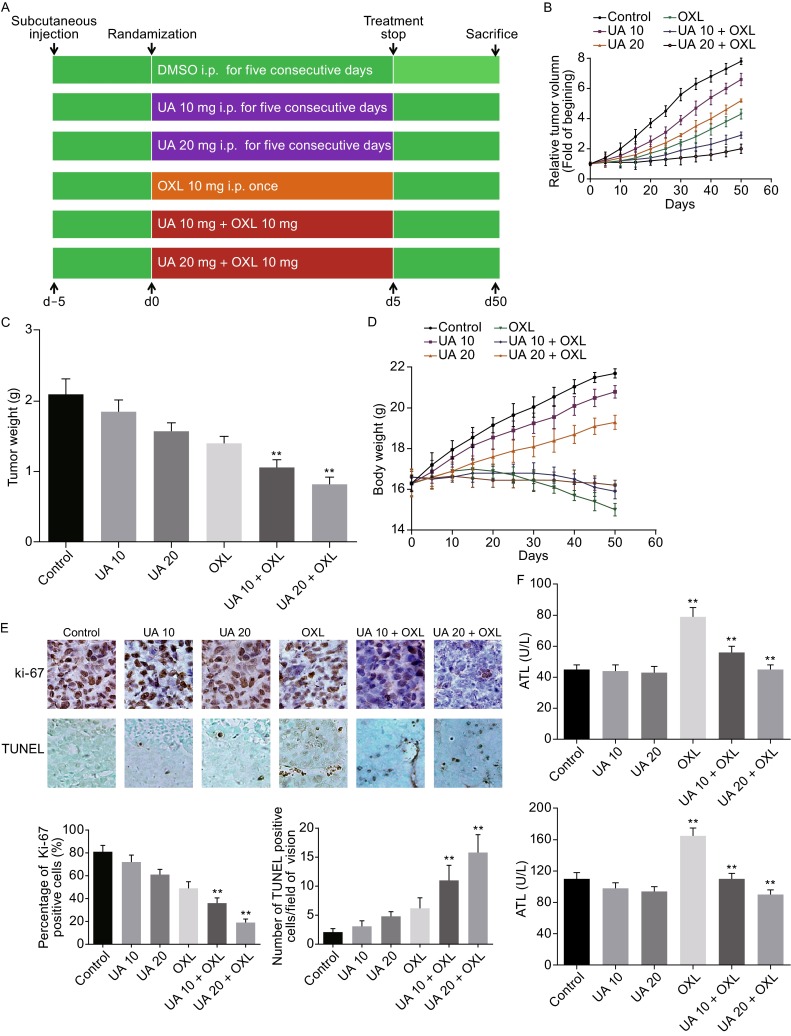
**Ursolic acid enhanced the ability of oxaliplatin in inhibiting the growth of implanted human xenografts in nude mice and induced apoptosis in tumor tissues**. (A) Schematic representation of the experimental protocol. (B) Tumor volumes measured during the course of the experiment. Data were expressed as fold changes compared to the control group for each time point. (C) Average tumor weight of each group weighed on the last day of the experiment. *, *P* < 0.05; **, *P* < 0.01 over vehicle controls. (D) Body weights of mice were assessed. (E) Immunohistochemistry analysis of Ki-67 and TUNEL positive cells in tumor tissues of each group. Quantitative analysis was performed for each method and the results were shown, respectively. (F) Serum ALT and AST levels were assessed. Ursolic acid alone (10 or 20 mg/kg) had no effect on liver function. Oxaliplatin impaired liver function, which was mollified in the presence of ursolic acid. **, *P* < 0.01

In view of the above *in vitro* data, we further tested the effects of this combination strategy *in vivo*. We established a SW620 xenograft mouse model and mice were randomized to six groups with different treatment strategies (Fig. 4A). We observed a tumor inhibitory role of ursolic acid or oxaliplatin treatment alone, with a comparable effect of ursolic acid at 20 mg/kg and oxaliplatin at 10 mg/kg. Noticeably, the mice treated with both ursolic acid and oxaliplatin showed the strongest tumor inhibition (Fig. 4B).

At the end of the experiment all tumors were collected and weighed. We found that ursolic acid and oxaliplatin alone significantly decreased tumor weight compared to the control group (*P* < 0.05; Fig. 4C). As expected, the combination treatment (group 5 and 6) achieved a much significant suppression compared to the control group (*P* < 0.01; Fig. 4C). Additionally, combination strategy succeeded in maintaining the body weight of mice (Fig. 4D), suggesting an improved toxicity of this strategy.

As a proliferation indicator, Ki-67 was reduced by both ursolic acid and oxaliplatin, alone or in combination (Fig. 4E). Moreover, increased tumor apoptosis was also detected by TUNEL assays. The combination treatment with ursolic acid and oxaliplatin resulted in increased tumor cell apoptosis compared to the control group (*P* < 0.01; Fig. 4E), which was correlated with the tumor burden observed at the end of the experiment.

Since it is reported that oxaliplatin would cause liver damage (Morris-Stiff et al., 2008; Nordlinger et al., 2013), we tested alanine aminotransferase (ALT) and aspartate aminotransferase (AST) levels in mice serum as indicators for liver function. Oxaliplatin significantly induced ALT and AST elevation, which was improved when ursolic acid was present (Fig. 4F).

### Ursolic acid and oxaliplatin suppressed phosphorylation of ERK1/2, AKT, and NF-κB in tumor tissues

**Figure 5:**
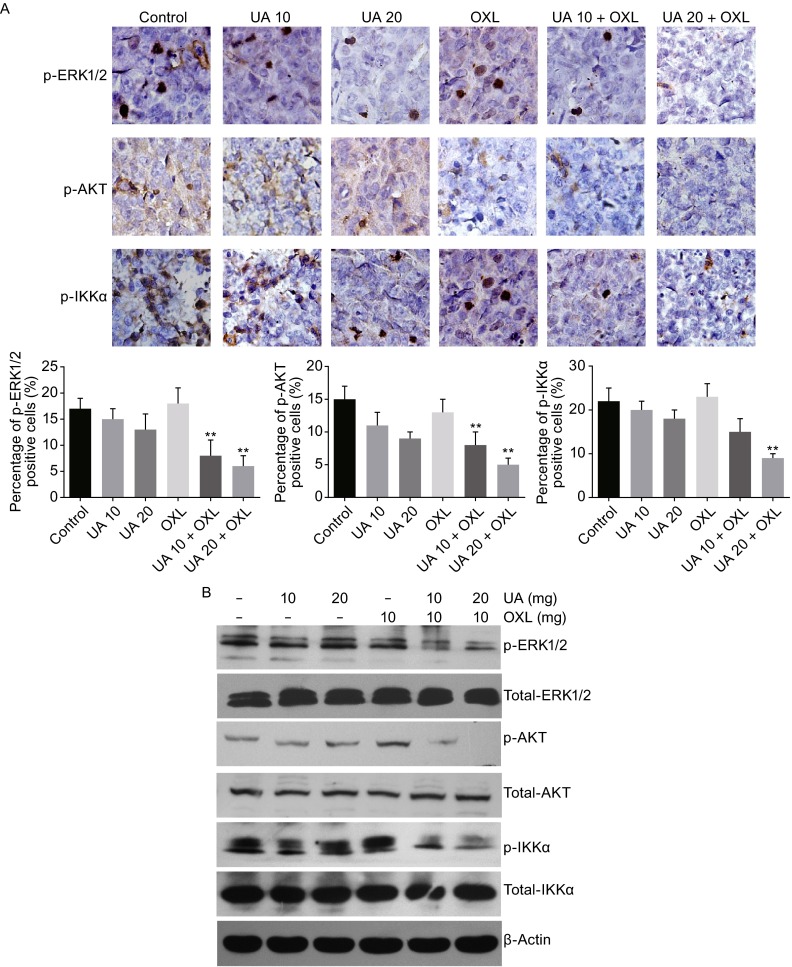
**Ursolic acid in combination with oxaliplatin suppressed phosphorylation of ERK1/2, AKT, and NF-κB in tumor tissues and it potentially protected liver function of the mice**. (A) Immunohistochemistry analysis of SW620 xenografts showed significantly decreased phosphorylation of ERK1/2, AKT, and IKKα in the combination group compared to either control or single oxaliplatin treated groups. Quantitative analysis was performed for each method and the results were shown, respectively. **, *P* < 0.01. (B) Western blot analysis showed that 10 or 20 mg/kg ursolic acid combined with 10 mg/kg oxaliplatin significantly inhibited the phosphorylation of ERK1/2, AKT, and IKKα in tumor tissues compared to either the control or the single oxaliplatin treated groups

To confirm the mechanisms by which ursolic acid with or without oxaplatin inhibited CRC we had identified *in vitro*, the xenograft tumor tissues were harvested and immunohistochemistry was performed. While a mild decrease of p-ERK1/2, p-AKT, and p-IKKα was observed on the xenograft samples from mice treated with ursolic acid alone, oxaliplatin alone did not seem to have obvious effect on the phosphorylation status of these three molecules tested (Fig. 5A). However, when combined with either 10 or 20 mg/kg of ursolic acid, the phosphorylation of ERK1/2, AKT, and IKKα in tumors was dramatically reduced (*P* < 0.01). Immunoblotting was further performed and supported that the combination treatment had a pronounced suppression of p-ERK1/2, p-AKT, and p-IKKα in the tumor samples (Fig. 5B). Collectively, these *in vivo* data strongly demonstrated that ursolic acid combined with oxaliplatin synergistically inhibited the growth of SW620 xenografts, probably via inactivating the MAPK, PI3K/AKT, and NF-κB signaling pathways.

## DISCUSSION

Keeping in mind that the cumulative dose of oxaliplatin administered to patients is the key factor of oxaliplatin-related adverse events, we herein investigated whether ursolic acid could enable a suboptimal use of oxaliplatin without compromise of its efficacy. Our results revealed that ursolic acid was capable of synergistically enhancing the chemotherapeutic effects of oxaliplatin in CRC both *in vitro* and *in vivo*. In particular, the cytotoxicity of 0.4 μmol/L oxaliplatin in the presence of 10 μmol/L ursolic acid was comparable to that of 0.7 μmol/L oxaliplatin. The effect of this combination strategy was repeatedly observed using various experimental methods and therefore was convincing. Our xenograft mouse model also showed that ursolic acid combined with oxaliplatin achieved the best tumor control effects among the strategies tested. To our best knowledge, this is the first report showing that ursolic acid could synergistically enhance the therapeutic effects of oxaliplatin in CRC both *in vitro* and *in vivo*.

Notably, it has been shown that oxaliplatin-induced peripheral neurotoxicity may be mediated by early p38 and ERK1/2 activation in the dorsal root ganglia neurons (Hector et al., 2001; Scuteri et al., 2009). In support of this conclusion, another study demonstrated that a MEK1/2 inhibitor PD0325901 was able to suppress the oxaliplatin-induced neuropathy and synergistically potentiated the tumor suppressive effects of oxaliplatin in a colon carcinoma allograft mouse model (Tsubaki et al., 2015). Considering its strong inhibitory effects against multiple kinases activation in CRC cells observed in our study, it seems plausible to hypothesize that ursolic acid may also play a neuroprotective role against oxaliplatin-induced neuropathy while enhancing its cytotoxicity against tumor cells. Due to the limitations of our xenograft mouse model, it is difficult to directly assess the incidence of neuropathy and the effect of ursolic acid on this adverse event. Further studies may be needed to establish specific mouse models to better evaluate the potential neuroprotective role of ursolic acid against oxaliplatin-induced neurotoxicity.

Hepatic complications were also clinically relevant adverse events following preoperative chemotherapy with oxaliplatin for hepatic colorectal metastases (Morris-Stiff et al., 2008; Nordlinger et al., 2013). In our mouse model, only a single dose of oxaliplatin caused a significant increase in serum ALT and AST levels, which was restored by addition of ursolic acid in a dose-dependent manner, strongly suggesting its liver protective effect. In fact, we observed that mice treated with oxaliplatin combined with ursolic acid showed better life quality compared to mice treated with oxaliplatin alone, which can be partially explained by decreased tumor burden, better liver function, and restored body weight of these mice. The liver protective role of ursolic acid was also previously reported in an ethanol-mediated experimental rat model by its antioxidant activity (Li et al., 2014; Saravanan et al., 2006). Therefore, ursolic acid may be a promising compound to be administered in combination with oxaliplatin or possibly other chemotherapeutic drugs to protect patients’ liver function.

It has long been known that the MAPK, PI3K/AKT, and NF-κB signaling pathways play key roles in controlling a wide variety of cellular functions such as cell proliferation, differentiation, and regulation of apoptosis (Hassanzadeh, 2011; Hatzivassiliou et al., 2013; Temraz et al., 2015). Aberrant activation of these pathways can lead to tumorigenesis and confer drug resistance. Activating mutations of KRAS and BRAF are commonly occurred in CRC and have been identified as predictors of resistance to EGFR monoclonal antibodies such as cetuximab and were associated with poor survival (Bertotti et al., 2015; De Roock et al., 2010). Co-existing mutations in PI3K/AKT pathway further confers resistance to MEK inhibitor treatment in KRAS mutant CRC (Shimizu et al., 2012). Due to the crosstalk among signaling pathways, cancer cells treated with a single target drug inevitably activate alternative pathways as escape mechanisms to overcome the blockage and therefore the effectiveness of these drugs. In our study, we did find that SW480 and SW620 cells harboring *KRAS*^*G12V*^ mutations and LoVo cells harboring *KRAS*^*G13D*^ mutations showed resistance to single cetuximab treatment (Data not shown). RKO cells harboring both *BRAF*^*V600E*^ and *PIK3CA*^*H1047R*^ mutations showed resistance to single cetuximab or MEK inhibitor treatment (Data not shown). However, all the four cell lines were sensitive to ursolic acid treatment. This observations were in line with the current notion that rational combination of targeted treatments to circumvent, reverse, or even preclude resistance are necessary for optimum use of molecular targeted therapies in cancer (De Roock et al., 2011). Ursolic acid, a compound proven to target multiple signaling pathways (Leelawat et al., 2009), may therefore serve as a promising candidate to effectively kill resistant cancer cells either individually or by combining with other targeted drugs.

With regard to drug resistance specific to oxaliplatin in cancers, some evidence implicated potential roles of NF-κB and PI3K/AKT signaling pathways. It has been shown that the sensitivity of colorectal cancer cells to oxaliplatin-induced death is adversely affected by elevated NF-κB activity (Shimizu et al., 2012). In cholangiocarcinoma cells, activation of PI3K/AKT may cause resistance to oxaliplatin-induced cytotoxicity (Hayward et al., 2004). Moreover, aberrant expression of anti-apoptotic proteins such as Bcl-xL, Bcl-2, and survivin were observed in human CRC cell lines with acquired resistance to oxaliplatin (Hayward RL, et al., 2004). In our study, we observed pronounced suppression of the PI3K/AKT and NF-κB signaling pathways as well as Bcl-xL, Bcl-2, and survivin by ursolic acid, which made it a promising candidate for potential treatment of oxaliplatin-resistant cancers. However, further investigations are needed to validate this hypothesis.

In conclusion, we proved a synergistic effect of ursolic acid and oxaliplatin against CRC. This effect was associated with the inhibition of multiple kinase pathways including MAPK, PI3K/AKT, and NF-κB signaling pathways. This strategy is promising in better tumor control and improved adverse events.

## MATERIALS AND METHODS

### Compounds and antibodies

Ursolic acid was purchased from Sigma (MO, USA), dissolved in dimethyl sulfoxide, aliquoted and stored at −20°C. Oxaliplatin was purchased from Minsheng-Sanofi (Hangzhou, China). Cetuximab manufactured by Merck chemicals (Darmstadt, Germany). The primary antibodies used in the study including phospho-IKKα, IKKα, phospho-IκBα, IκBα, phospho-NF-κB p65, p65 were obtained from ABCAM company (Cambridge, UK). Other primary antibodies including phospho-B-Raf, B-Raf, Phospho-Mek1/2, Mek1/2, Phospho-ERK1/2, ERK1/2, phospho-AKT, AKT, phospho-JNK, JNK and phospho-p38 kinases, P38 kinases, caspase-3, caspase-8, caspase-9, Bcl-2, Bcl-xL, Survivin were purchased from Cell Signal Company (MA, USA). The secondary antibodies including HRP-conjugated anti-rabbit IgG or anti-mouse IgG were purchased from Santa Cruz Biotechnology (CA, USA).

### Cell lines and culture conditions

The four human CRC cell lines SW480, SW620, LoVo, and RKO were purchased from American Type Culture Collection (ATCC, Manassas, VA, USA). SW480 and SW620 cells were cultured in Leibovitz’s L-15 medium, LoVo cells were cultured in F-12K medium and RKO cells were cultured in RPMI-1640 medium which were all supplemented with 10% FBS, 100 U/mL penicillin, and 100 µg/mL streptomycin.

### Cell proliferation assay

The effect of ursolic acid or oxaliplatin alone or in combination on cell proliferation was determined by measuring the mitochondria dehydrogenase activity, using MTT as the substrate. The absorbance was measured at 570 nm using an MRX Revelation 96-well multiscanner (Dynex Technologies, Chantilly, VA, USA).

### Apoptosis assay

To detect cell apoptosis caused by ursolic acid and oxaliplatin alone or in combination, we used an annexin V-FITC/propidium iodide staining kit (Sigma, St. Louis, MO, USA) as described previously (Shan et al., 2009). Briefly, 1 × 10^6^ cells were treated with indicated doses of ursolic acid and/or oxaliplatin for 48 h. Cells were harvested and stained with assay reagents and cell apoptosis was determined by flow cytometry using a FACS Caliber (Becton Dickinson, CA, USA).

Alternatively, the apoptotic cells were determined by propidium iodide staining as described previously. Briefly, 1 × 10^6^ cells were harvested and washed in PBS, then fixed in 75% ethanol for 24 h at 4°C. After three-time washes with ice-cold PBS, cells were resuspended in 1 mL of PBS solution with 40 μg of propidium iodide (Sigma) and 100 μg of RNase A (Sigma) for 30 min at 37°C. Samples were then analyzed for their DNA content by a FACS Caliber. The Sub-G_1_ population represented the apoptotic cells.

### Detection of mitochondrial membrane potential

Mitochondrial membrane potential was measured according to the manufacturer’s instructions. Briefly, one million of SW620 cells were collected after treating with indicated doses of ursolic acid and/or oxaliplatin for 48 h. Cells were resuspended in 1 mL PBS containing 1 mmol/L Rhodamine 123 for 30 min and were then measured by flow cytometry using a FACS Caliber (Becton Dickinson, CA, USA).

### Terminal deoxynucleotidyl transferase dUTP nick-end labeling assay (TUNEL)

The TUNEL assay was used to evaluate cell apoptosis according to the manufacture’s instruction (Calbiochem, Darmstadt, Germany). Briefly, a half million of cells were plated in complete medium and incubated with different concentrations of ursolic acid alone or in combination with oxaliplatin for 48 h. Cells were harvested, fixed in 10% buffered neutral formalin, dehydrated, and embedded in paraffin. Similarly, tumor samples were also collected and fixed in 10% formalin before embedded in paraffin and 4 μm thick sections were prepared and stained for TUNEL analysis. Slides were then counterstained with hematoxylin solution and were viewed under a fluorescence microscope (Olympus B*41, Tokyo, Japan). Apoptotic cells were quantified and expressed as percentage of total number of cells viewed in 10 randomly selected fields at 400× magnifications.

### Hoechst 33342 staining

SW620 cells were plated and treated with ursolic acid alone or in combination with oxaliplatin for 48 h. Cells were then collected and stained with FLICA reagent (Immunohistochemistry Technologies LLC, Bloomington, MN, USA) and incubated for 45 min at 37°C. Hoechst dye (Acros Organics, Belgium) at a final concentration of 10 mg/mL was added and incubated for an additional 10 min. Cells were then washed and visualized under a Zeiss fluorescence microscope with UV and rhodamine filters.

### Combination effects of ursolic acid and oxaliplatin

Drug combination effects of ursolic acid and oxaliplatin were evaluated using MTT assay in all the four CRC cell lines. Briefly, a total of one million of cells were plated in triplets and were treated with either ursolic acid alone (0, 1.25, 2.5, 5, 10, 20, 40, 60, 80, and 100 μmol/L) or oxaliplatin alone (0, 0.125, 0.25, 0.5, 1, 2, 4, 6, 8, and 10 μmol/L) or ursolic acid in combination with oxaliplatin at fixed ratios. After incubation for 48 h in the dark at 37°C, MTT lysis buffer was added as described previously. The *in vitro* drug interactions were analyzed according to the method previously described (Chou, 2006). The combination index (CI) was calculated according to the -isobologram equation: CI = D1/Dx1 + D2/Dx2. (*D*)1 and (*D*)2 in the numerators are the doses of D1 (ursolic acid) and D2 (oxaliplatin) in combination that generate *x*% inhibition whereas (Dx)1 and (Dx)2 in the denominators are the doses of D1 and D2 that also give *x*% inhibition individually. CI = 1 indicates an additive effect, CI < 1 indicates a synergistic effect and CI > 1 indicates an antagonistic effect.

### Western blot analysis

Western blot analysis was performed as descried before with minor adjustments (Shan et al., 2009). Briefly, 5 × 10^5^ cells were incubated in the presence or absence of ursolic acid or in combination with oxaliplatin for 48 h and were lysed in a sample buffer supplemented with protease inhibitor cocktail (Pierce, Rockford, IL, USA). Specifically, nuclear and cytoplasmic protein was extracted using a nuclear and cytoplasmic protein extraction kit (P0028, Beyotime biotechnology, Shanghai, China). Protein concentration was measured by BCA protein assay (Pierce). The protein was loaded into a 10% SDS-PAGE and transferred to a PVDF membrane. The membrane was then incubated with the respective primary and secondary antibodies. Signals were detected by a chemiluminescence kit (Pierce).

### SW620 xenograft nude mouse model

The study protocol was approved by the ethic committee of the Second Affiliated Hospital, Zhejiang University School of Medicine. SW620 cells (1 × 10^7^) were injected subcutaneously into the right flank area of 6–8-week-old female nude mice. Five days after inoculation, animals were divided randomly into six groups with six mice per group. Animals in the control group (Group 1) were treated with sterile DMSO via intraperitoneal injection for five consecutive days. Animals in Group 2 and 3 were administered with 10 mg/kg or 20 mg/kg ursolic acid intraperitoneally for five consecutive days, respectively. Animals in the rest three groups (Group 4, 5, 6) were either given a single dose of 10 mg/kg oxaliplatin alone via intraperitoneal injection, or combined with 10 mg/kg or 20 mg/kg ursolic acid for five consecutive days, respectively. Mouse survival was monitored on the daily basis. Tumor growth and mouse body weight were assessed twice weekly. Tumor volume (mm^3^) was measured using a caliper and was calculated using the following equation (Chou, 2006): Tumor volume (mm^3^) = (length × width^2^) × 0.5. All mice were sacrificed at day 50 and 0.5 mL of orbital blood was obtained from each mouse. Blood samples were sent to the clinical laboratory for ALT and AST measurement. Additionally, tumors were harvested and weighed. Student’s *t*-test was used to compare tumor weight among different groups. Tumors were then separated into two halves. One half of the tumor tissues were immediately snap-frozened in liquid nitrogen and stored at −80°C for later examination of protein phosphorylation by SDS-PAGE and Western blot. The other half of the tumor tissues were fixed in 10% buffered neutral formalin, dehydrated, and embedded in paraffin for further immunohistochemical analysis.

### Immunohistochemistry

Immunohistochemical analysis was performed according to the manufacturer’s instructions. Briefly, slides were incubated with respective primary antibodies and corresponding secondary antibodies conjugated with horse radish peroxidase to detect the antigen-antibody reaction (Multisciences Biotech, Shanghai, China). Sections were then incubated with 3,3-diaminobenzidine as a chromogen for 5 min and were counterstained with hematoxylin. Slides were then washed in tap water, dehydrated, and mounted with glass coverslips. The same sections incubated with non-immunized serum were used as negative controls. All comparisons of staining intensities were made at 400-fold magnifications.

### Statistical analysis

All experiments were repeated at least three times and data are presented as means and standard deviation (SD) values. Prism 6 (GraphPad, SanDiego, CA, USA) was used to perform Statistical analysis. The Student’s *t*-test was used for comparison between groups and a value of *P* < 0.05 was considered statistically significant.

## References

[CR1] Achiwa Y, Hasegawa K, Udagawa Y (2013). Effect of ursolic acid on MAPK in cyclin D1 signaling and RING-type E3 ligase (SCF E3s) in two endometrial cancer cell lines. Nutr Cancer.

[CR2] Andre T, Boni C, Navarro M, Tabernero J, Hickish T, Topham C, Bonetti A, Clingan P, Bridgewater J, Rivera F (2009). Improved overall survival with oxaliplatin, fluorouracil, and leucovorin as adjuvant treatment in stage II or III colon cancer in the MOSAIC trial. J Clin Oncol.

[CR3] Avan A, Postma TJ, Ceresa C, Avan A, Cavaletti G, Giovannetti E, Peters GJ (2015). Platinum-induced neurotoxicity and preventive strategies: past, present, and future. Oncologist.

[CR4] Bertotti A, Papp E, Jones S, Adleff V, Anagnostou V, Lupo B, Sausen M, Phallen J, Hruban CA, Tokheim C (2015). The genomic landscape of response to EGFR blockade in colorectal cancer. Nature.

[CR5] Checker R, Sandur SK, Sharma D, Patwardhan RS, Jayakumar S, Kohli V, Sethi G, Aggarwal BB, Sainis KB (2012). Potent anti-inflammatory activity of ursolic acid, a triterpenoid antioxidant, is mediated through suppression of NF-κB, AP-1 and NF-AT. PLoS One.

[CR6] Chen W, Zheng R, Zeng H, Zhang S, He J (2015). Annual report on status of cancer in China, 2011. Chin J Cancer Res.

[CR7] Chou TC (2006). Theoretical basis, experimental design, and computerized simulation of synergism and antagonism in drug combination studies. Pharmacol Rev.

[CR8] Ciombor KK, Wu C, Goldberg RM (2015). Recent therapeutic advances in the treatment of colorectal cancer. Ann Rev Med.

[CR9] De Roock W, Claes B, Bernasconi D, De Schutter J, Biesmans B, Fountzilas G, Kalogeras KT, Kotoula V, Papamichael D, Laurent-Puig P (2010). Effects of KRAS, BRAF, NRAS, and PIK3CA mutations on the efficacy of cetuximab plus chemotherapy in chemotherapy-refractory metastatic colorectal cancer: a retrospective consortium analysis. Lancet Oncol.

[CR10] De Roock W, De Vriendt V, Normanno N, Ciardiello F, Tejpar S (2011). KRAS, BRAF, PIK3CA, and PTEN mutations: implications for targeted therapies in metastatic colorectal cancer. Lancet Oncol.

[CR11] Ebert SM, Dyle MC, Bullard SA, Dierdorff JM, Murry DJ, Fox DK, Bongers KS, Lira VA, Meyerholz DK, Talley JJ (2015). Identification and small molecule inhibition of an activating transcription factor 4 (ATF4)-dependent pathway to age-related skeletal muscle weakness and atrophy. J Biol Chem.

[CR12] Gao N, Cheng S, Budhraja A, Gao Z, Chen J, Liu EH, Huang C, Chen D, Yang Z, Liu Q (2012). Ursolic acid induces apoptosis in human leukaemia cells and exhibits anti-leukaemic activity in nude mice through the PKB pathway. Br J Pharmacol.

[CR13] Hassanzadeh P (2011). Colorectal cancer and NF-κB signaling pathway. Gastroenterol Hepatol Bed Bench.

[CR14] Hatzivassiliou G, Haling JR, Chen H, Song K, Price S, Heald R, Hewitt JF, Zak M, Peck A, Orr C (2013). Mechanism of MEK inhibition determines efficacy in mutant KRAS- versus BRAF-driven cancers. Nature.

[CR15] Hayward RL, Macpherson JS, Cummings J, Monia BP, Smyth JF, Jodrell DI (2004). Enhanced oxaliplatin-induced apoptosis following antisense Bcl-xl down-regulation is p53 and Bax dependent: Genetic evidence for specificity of the antisense effect. Mol Cancer Ther.

[CR16] He W, Shi F, Zhou ZW, Li B, Zhang K, Zhang X, Ouyang C, Zhou SF, Zhu X (2015). A bioinformatic and mechanistic study elicits the antifibrotic effect of ursolic acid through the attenuation of oxidative stress with the involvement of ERK, PI3K/Akt, and p38 MAPK signaling pathways in human hepatic stellate cells and rat liver. Drug Des Dev Ther.

[CR17] Hector S, Bolanowska-Higdon W, Zdanowicz J, Hitt S, Pendyala L (2001). In vitro studies on the mechanisms of oxaliplatin resistance. Cancer Chemother Pharmacol.

[CR18] Leelawat K, Narong S, Udomchaiprasertkul W, Leelawat S, Tungpradubkul S (2009). Inhibition of PI3K increases oxaliplatin sensitivity in cholangiocarcinoma cells. Cancer Cell Int.

[CR19] Li J, Liang X, Yang X (2012). Ursolic acid inhibits growth and induces apoptosis in gemcitabine-resistant human pancreatic cancer via the JNK and PI3K/Akt/NF-κB pathways. Oncol Rep.

[CR20] Li S, Liao X, Meng F, Wang Y, Sun Z, Guo F, Li X, Meng M, Li Y, Sun C (2014). Therapeutic role of ursolic acid on ameliorating hepatic steatosis and improving metabolic disorders in high-fat diet-induced non-alcoholic fatty liver disease rats. PLoS One.

[CR21] Martinez-Balibrea E, Martinez-Cardus A, Gines A, Ruiz DPV, Moutinho C, Layos L, Manzano JL, Buges C, Bystrup S, Esteller M (2015). Tumor-related molecular mechanisms of oxaliplatin resistance. Mol Cancer Ther.

[CR22] Morris-Stiff G, Tan YM, Vauthey JN (2008). Hepatic complications following preoperative chemotherapy with oxaliplatin or irinotecan for hepatic colorectal metastases. Eur J Surg Oncol.

[CR23] Nordlinger B, Sorbye H, Glimelius B, Poston GJ, Schlag PM, Rougier P, Bechstein WO, Primrose JN, Walpole ET, Finch-Jones M (2013). Perioperative FOLFOX4 chemotherapy and surgery versus surgery alone for resectable liver metastases from colorectal cancer (EORTC 40983): long-term results of a randomised, controlled, phase 3 trial. Lancet Oncol.

[CR24] Prasad S, Yadav VR, Kannappan R, Aggarwal BB (2011). Ursolic acid, a pentacyclin triterpene, potentiates TRAIL-induced apoptosis through p53-independent up-regulation of death receptors: evidence for the role of reactive oxygen species and JNK. J Biol Chem.

[CR25] Prasad S, Yadav VR, Sung B, Reuter S, Kannappan R, Deorukhkar A, Diagaradjane P, Wei C, Baladandayuthapani V, Krishnan S (2012). Ursolic acid inhibits growth and metastasis of human colorectal cancer in an orthotopic nude mouse model by targeting multiple cell signaling pathways: chemosensitization with capecitabine. Clin Cancer Res.

[CR26] Saravanan R, Viswanathan P, Pugalendi KV (2006). Protective effect of ursolic acid on ethanol-mediated experimental liver damage in rats. Life Sci.

[CR27] Scuteri A, Galimberti A, Maggioni D, Ravasi M, Pasini S, Nicolini G, Bossi M, Miloso M, Cavaletti G, Tredici G (2009). Role of MAPKs in platinum-induced neuronal apoptosis. Neurotoxicology.

[CR28] Shan JZ, Xuan YY, Zheng S, Dong Q, Zhang SZ (2009). Ursolic acid inhibits proliferation and induces apoptosis of HT-29 colon cancer cells by inhibiting the EGFR/MAPK pathway. J Zhejiang Univ Sci B.

[CR29] Shan JZ, Xuan YY, Ruan SQ, Sun M (2011). Proliferation-inhibiting and apoptosis-inducing effects of ursolic acid and oleanolic acid on multi-drug resistance cancer cells in vitro. Chin J Integr Med.

[CR30] Shanmugam MK, Rajendran P, Li F, Nema T, Vali S, Abbasi T, Kapoor S, Sharma A, Kumar AP, Ho PC (2011). Ursolic acid inhibits multiple cell survival pathways leading to suppression of growth of prostate cancer xenograft in nude mice. J Mol Med.

[CR31] Shanmugam MK, Dai X, Kumar AP, Tan BK, Sethi G, Bishayee A (2013). Ursolic acid in cancer prevention and treatment: molecular targets, pharmacokinetics and clinical studies. Biochem Pharmacol.

[CR32] Shimizu T, Tolcher AW, Papadopoulos KP, Beeram M, Rasco DW, Smith LS, Gunn S, Smetzer L, Mays TA, Kaiser B (2012). The clinical effect of the dual-targeting strategy involving PI3K/AKT/mTOR and RAS/MEK/ERK pathways in patients with advanced cancer. Clin Cancer Res.

[CR33] Shishodia S, Majumdar S, Banerjee S, Aggarwal BB (2003). Ursolic acid inhibits nuclear factor-kappaB activation induced by carcinogenic agents through suppression of IκBα kinase and p65 phosphorylation: correlation with down-regulation of cyclooxygenase 2, matrix metalloproteinase 9, and cyclin D1. Cancer Res.

[CR34] Siegel R, Naishadham D, Jemal A (2013). Cancer statistics, 2013. CA Cancer J Clin.

[CR35] Temraz S, Mukherji D, Shamseddine A (2015). Dual inhibition of MEK and PI3K Pathway in KRAS and BRAF mutated colorectal cancers. Int J Mol Sci.

[CR36] Tsubaki M, Takeda T, Tani T, Shimaoka H, Suzuyama N, Sakamoto K, Fujita A, Ogawa N, Itoh T, Imano M (2015). PKC/MEK inhibitors suppress oxaliplatin-induced neuropathy and potentiate the antitumor effects. Int J Cancer.

[CR37] Wozniak L, Skapska S, Marszalek K (2015). Ursolic acid–a pentacyclic triterpenoid with a wide spectrum of pharmacological activities. Molecules.

[CR38] Xavier CP, Lima CF, Preto A, Seruca R, Fernandes-Ferreira M, Pereira-Wilson C (2009). Luteolin, quercetin and ursolic acid are potent inhibitors of proliferation and inducers of apoptosis in both KRAS and BRAF mutated human colorectal cancer cells. Cancer Lett.

[CR39] Xiang L, Chi T, Tang Q, Yang X, Ou M, Chen X, Yu X, Chen J, Ho RJ, Shao J (2015). A pentacyclic triterpene natural product, ursolic acid and its prodrug US597 inhibit targets within cell adhesion pathway and prevent cancer metastasis. Oncotarget.

